# Drop Dissolution Intensified by Acoustic Levitation

**DOI:** 10.3390/mi15060805

**Published:** 2024-06-20

**Authors:** Jan-Paul Ruiken, Jörn Villwock, Matthias Kraume

**Affiliations:** Department of Chemical and Process Engineering, Technische Universität Berlin, Ackerstr. 76, 13355 Berlin, Germany; ruiken@tu-berlin.de (J.-P.R.); matthias.kraume@tu-berlin.de (M.K.)

**Keywords:** acoustic levitation, drops, liquid–liquid system, dissolution, binary mass transfer

## Abstract

Acoustic levitation can provide significant benefits for many fundamental research questions. However, it is important to consider that the acoustic field influences the measurement environment. This work focuses on the dissolution of immobilised drops using acoustic levitation in liquid–liquid systems. Previous work demonstrated that the acoustic field of standing waves impacts mass transfer by affecting the spread of dissolved substances in the continuous phase in two distinct ways: (I) solutes may either pass through nodal planes of the standing waves or (II) not pass. The binary systems examined for case (I) are 1-hexanol–water and 1-butanol–water, and for case (II), n-butyl acetate–water and toluene–water. This work quantifies the intensification effect of acoustic levitation on dissolution for the two types of behaviour, by comparing them with reference measurements of mechanically attached dissolving drops. The system was designed to ensure minimal intensification. The minimum intensification of mass transfer for levitating drops in the used setup of case (I) was 25%, and for case (II), it was 65%, both increasing with decreasing surface-equivalent diameter. With this understanding, acoustic levitation can be used more accurately in the field of mass transfer studies.

## 1. Introduction

The dissolution of immobile single drops without a superimposed flow field is governed by diffusion and natural convection. Here, natural convection results from buoyancy-driven flow caused by density gradients due to concentration changes in the continuous phase. State-of-the-art methods for investigating this mechanism typically involve mechanical contact to surfaces for immobilisation, such as sessile drops attached to walls or drops attached to capillaries. The shape and size of a drop in the first method are strongly influenced by the surface tension and the resulting contact angle between the drop and the wall material [1,2,3,4]. As a result, it is difficult to obtain accurate observations of immobilised spherical drops. The second method has been used for several investigations concerning mass transfer during [5,6,7] and after drop formation [8,9,10]. The surface tension limits the maximum diameter of the drop. Furthermore, when measuring ternary or multi-component systems, mass transfer between the inner capillary volume and the drop volume can interfere with measurements. In such cases, the transferring component has an additional and undefined reservoir within the capillary. Contact with a capillary can even trigger effects caused by gradients in interfacial tension, such as Marangoni convection [11].

To overcome some of the previously stated challenges, a low-impact acoustic levitation system in a liquid–liquid environment is used for further investigations. The developed system was reported recently in [12]. The ability to immobilise a drop for several days allows non-invasive optical measurements and can be used for a variety of fundamental research applications. Acoustic levitation can have strong advantages over other drop immobilisation methods. However, it can also have some undesired side effects depending on the application. The objective of this work was to further investigate the characteristics of acoustic levitation in liquid–liquid systems. The influence of acoustic levitation on mass transfer has already been discussed in [12] and will now be quantified for dissolving drops in binary chemical systems.

## 2. Materials and Methods

The setup for low-impact acoustic levitation was used to measure immobilised dissolving single drops. The acoustic levitation measurements (ALMs) were conducted following the procedure obtained in [12]. Alternations to the settings would modify the acoustic environment, potentially leading to different results. Four chemical systems with two distinct acoustic behaviours and varying drop dissolution times ranging from minutes to days were investigated. The propagation of inhomogeneously dissolved solutes in the continuous phase can behave in the spread differently: it can either (I) pass through nodal planes of the standing waves or (II) not pass through them. The effect of standing waves on dissolution was quantified by reference measurements carried out using glass rod measurements (GRMs). The GRMs simulate the undisturbed dissolution of immobilised drops.

Three of the dispersed phases investigated were based on the standard test systems for extraction of the European Federation of Chemical Engineering [13]: 1-butanol, n-butyl acetate, and toluene. The investigation also included the dispersed phase of 1-hexanol to have two systems of each observed spread behaviour. Ultrapure water formed the continuous phase. Before every experiment, all phases were degassed. In addition to the type of behaviour, the systems differed primarily in interfacial tension and aqueous solubility. The chemical properties of the pure substances used are given in Table 1.

**Figure 1:**
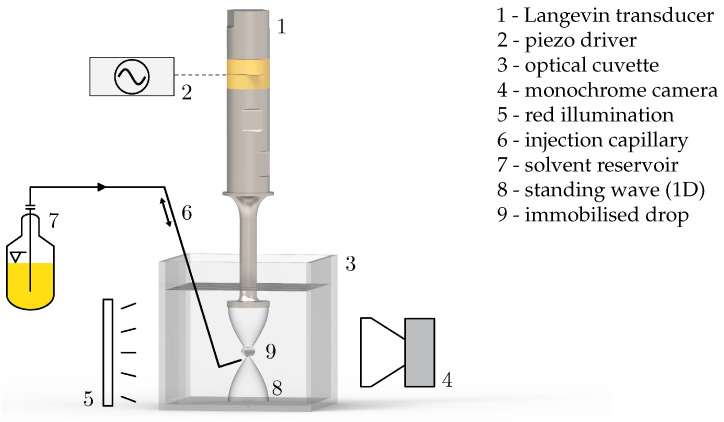
Schematic visualisation of the acoustic levitation setup. The standing wave is shown as one-dimensional, although it has a three-dimensional shape. A more detailed version can be found in [12].

**Dissolving drops, acoustically levitated**: The setup used is shown in Figure 1. To generate the standing wave for acoustic levitation, a Langevin transducer with a plate tip was precisely positioned in the optical cuvette and driven at a frequency of 66.5 kHz with a power input of ≈1 W. The cuvette was then filled with ≈100 mL of degassed continuous phase to a precise level. The degassing of all liquids was crucial to prevent strong vibrations of the drop caused by cavitation and degassing from the acoustic field. The drop was automatically injected with a capillary into the system close to the anti-node of the standing wave, where it was levitated for dissolution. The process was monitored by a monochromatic camera to acquire volume and shape data (see Video S1, Supplementary Material). The second optical axis was used for rainbow schlieren deflectometry observations developed by [15,16]. This optical method visualises gradients in the refractive index within a measuring volume. The concentration of a substance in a liquid determines the refractive index, which can be visualised indirectly with this method. The setup used is described in more detail in [12].

**Figure 2:**
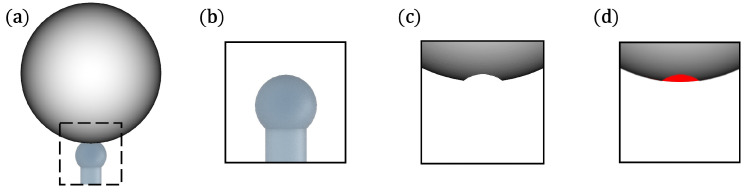
Glass rod measurements. (**a**) Acquired image of drop attached to glass rod. (**b**) Glass rod at the end of the measurement. (**c**) Images (**a**,**b**) for the extraction of the dispersed volume. (**d**) Free drop.

**Dissolving drops, on a glass rod as reference measurements**: To quantify the raw dissolution caused by diffusion and density-driven natural convection, a drop of a given diameter was first levitated in the system. A glass rod (Ø 0.5 mm), concentrically placed under the drop, was then moved up to touch and attach to the drop. The intensity of the acoustic field was decreased slowly to zero to be able to attach drops of maximum diameter. The maximum diameter differs with the chemical system and varies with the interfacial tension with glass. The stabilising effect of buoyancy enabled the drop to remain in a concentric alignment with the glass rod (Figure 2a). The volume was recorded until the drop completely dissolved into the continuous phase (see Video S2, Supplementary Material). At the end of the measurement, the shape of the capillary was captured for a brief period (Figure 2b) for later volume acquisition.

The dispersed volume $V{\left(t\right)}_{GRM}$ and the interfacial area in contact with the continuous phase $A{\left(t\right)}_{GRM}$ were obtained from Figure 2c, assuming a drop symmetry along the z-axis.

## 3. Results and Discussions

The authors recently demonstrated that the standing wave affects the spread of a substance dissolved inhomogeneously in the continuous phase. Two contrary effects were identified [12]:

(I) For one group of substances, the spread was not affected by the nodal plane above the drop. However, regions of maximum acoustic particle velocity at the outer tip of the horn of the Langevin transducer could not be overcome;
(II) For the other group of substances, the nodal planes act as barriers.

**Figure 3:**
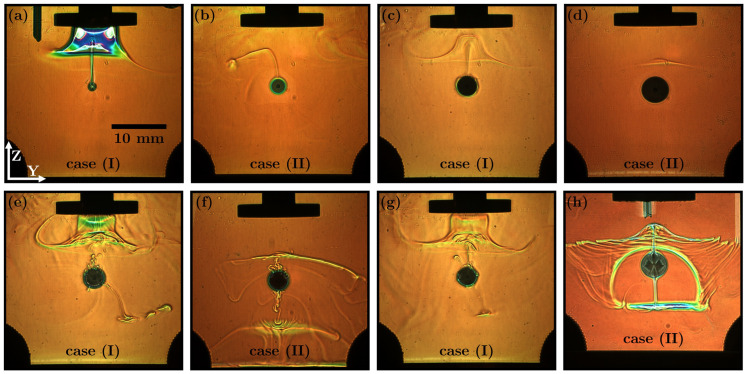
Rainbow schlieren deflectometry measurements [15,16] qualitatively indicating the concentration of the transferred substance of the binary systems: (**a**) 1-butanol; (**b**) n-butyl acetate; (**c**) 1-hexanol; and (**d**) toluene in water. Additional examples of the types of behaviour of some ternary systems that are not discussed further: (**e**) isopropanol–n-butyl acetate; (**f**) propylene carbonate–toluene; (**g**) ethanol–n-butyl acetate; and (**h**) morpholine–toluene in water. The images were obtained 8 min after injection.

These two types of behaviour measured by rainbow schlieren deflectometry are illustrated in Figure 3 and Videos S3 and S4 of the Supplementary Material. As the aqueous solubility of the dispersed phase decreased, the concentration and, hence, the intensity of the convective plume also decreased. For comparison, the factor between the aqueous solubilities of 1-hexanol (Figure 3a) and toluene (Figure 3d) was ≈170.

The two types of behaviour are clearly distinguishable optically. The different behaviours will affect the overall dissolution accordingly. Figure 3a–d show the four binary systems examined in more detail within this work. Four additional exemplary ternary systems, shown in Figure 3e–h, were introduced to address the behaviour under investigation in more detail. The first substance acted as the main transferred substance. The convective plumes were more intense and visible due to higher mass transfer rates and resulting concentration gradients.

With regard to case (II), the acoustic field could force a convective plume against its natural buoyancy to an opposite nodal plane of the drop (Figure 3f,h). In this case, the inhomogeneously dissolved substance was additionally pulled away from the concentration boundary layer. This additional pulling increased mass transfer and was not observed for the substances in case (I) in this distinct way. The two types of behaviour exhibited by the binary systems are examined quantitatively in more detail below.

**Figure 4:**
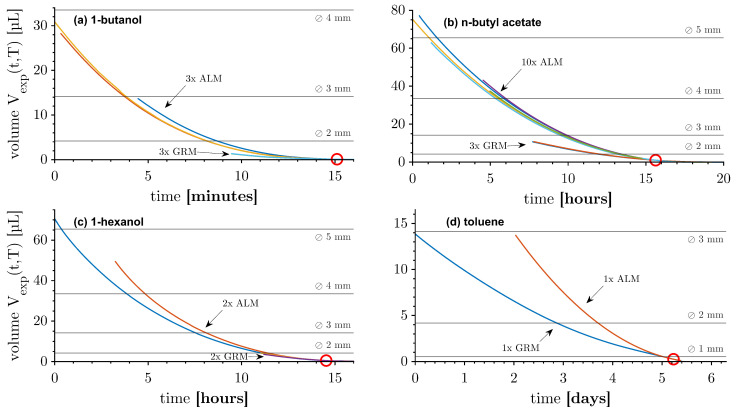
Volume of dissolving drops acoustically levitated (ALM) and glass rod measurements (GRMs) with varying dispersed phases in water: (**a**) 1-butanol; (**b**) n-butyl acetate; (**c**) 1-hexanol; and (**d**) toluene. The colours indicate different measurements, some measurements overlap and are not visible. The measurements of the substances are aligned on the abscissa to 0.01*$V_{\max}$ (red circle).

The dissolution behaviour of levitating drops and the drops attached to the glass rod is shown for 1-butanol in Figure 4a, for n-butyl acetate in Figure 4b, for 1-hexanol in Figure 4c, and for toluene in Figure 4d. The curves for each dispersed phase are horizontally aligned to a volume of 0.01*$V_{max}$, marked with a red circle. The surface tension between the dispersed phase and the glass rod limited the initial drop diameter of the GRM. The different aqueous solubilities resulted in dissolution times of the dispersed phase ranging from minutes to days. The dissolution of the ALM was intensified by the standing waves compared to the GRM for all chemical systems.

In the case of the GRM, the method had a negligible effect on the raw dissolution caused by diffusion and natural convection. The tip of the glass rod blocked a small interfacial area of the drop (Figure 2d, red area), which increased with decreasing drop diameter.

Upon initial observation, the measurements for acoustically levitating drops demonstrated moderate reproducibility for n-butyl acetate and 1-hexanol. The curves exhibited disparate shapes, and drops of the same size exhibited varying dissolution times. It is noteworthy that the acoustic levitation system lacked direct temperature control. Consequently, the continuous phase temperature of the measurements ranged from 15.3 to 23.5 °C, depending on the ambient room temperature (Table 2). The temperature exerted a direct impact on the aqueous solubility and diffusion coefficient of the dispersed phase and the properties of the acoustic environment. The operating point was subjected to a slight alternation due to the temperature, which also affected the overall dissolution.

The experimental volume curves $V_{\exp}\left(t,T\right)$ of the ALM and GRM serve as the foundation for all subsequent evaluations. The ALM drops were spherical, thus the equivalent diameter of a sphere of the same volume $d_{V}$ could be employed as a valid measure. In contrast, the GRM exhibited a non-spherical shape throughout the measurements, and with a part of the surface being obstructed by the glass rod. In the binary system with diffusion and natural convection occurring, the dissolution rate was mainly determined by the liquid–liquid surface area *A*. Therefore, the equivalent diameter of a sphere of the same area $d_{A}=\sqrt{A/\pi }$ was used to describe the size of the drop. To be consistent, this measure was also used for the ALM drops.

All measurements were evaluated, processed, and normalised to obtain the fitted volume flow curve ${\dot{V}}_{\mathrm{fit}}\left(d_{A}\right)$ in accordance with the guidelines set out in Equation (1). Although the volume flow is an atypical quantity for mass transport, it is here used as an unaltered signal and can easily be converted to a mass flow. In the following, the term “mass transfer” is used, although volume flow was used and measured:

(1) $$V_{\exp}\left(t,T\right)\overset{\mathrm{derivation}\;\mathrm{of}}{\underset{\mathrm{polyfit}\;n\;=\;9}{\to }}{\dot{V}}_{\exp}\left(t,T\right)\overset{d_{A}=\sqrt{A/\pi }}{\to }{\dot{V}}_{\exp}\left(d_{A},T\right)\overset{\mathrm{interpolating}}{\underset{\mathrm{to}\;T\;=\;19\;{}^{\circ}C}{\to }}{\dot{V}}_{\mathrm{inter}}\left(d_{A}\right)\overset{\mathrm{fit}\;\mathrm{to}}{\underset{\mathrm{power}\;\mathrm{function}}{\to }}{\dot{V}}_{\mathrm{fit}}\left(d_{A}\right),$$

where *t* denotes time, *T* denotes the temperature of the continuous phase, *A* denotes the liquid–liquid surface area of the drop, and $d_{A}$ denotes the surface area-equivalent diameter.

**Figure 5:**
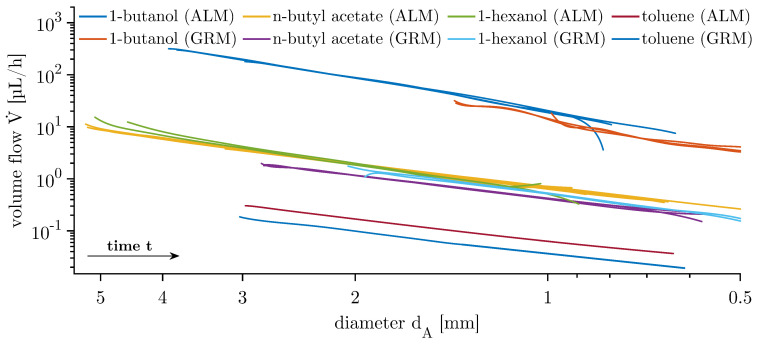
Volume flow ${\dot{V}}_{\exp}\left(d_{A}\right)$ (d→c) of dissolving drops showing acoustic levitation measurements (ALMs) and glass rod measurements (GRMs). The abscissa ($d_{A}$) is mirrored for a positive time trend.

As a preliminary step, the experimental volume curves $V_{\exp}\left(t,T\right)$ were all fitted by polynomial functions of the grade n=9 with residues of less than 0.1219 µL. As a next step in normalising the measurements to a specific temperature and averaging, the experimental volume flow curves ${\dot{V}}_{\exp}\left(t,T\right)$ were plotted against the surface area-equivalent diameter $d_{A}$ in Figure 5. This allowed for comparing the chemical systems with dissolution times of different scales. The dissolution speed, namely, the time dependency, of the drops could be read from the magnitude of the volume flow.

The slope of almost all curves in Figure 5, displayed with a log-log scale, is constant and can, therefore, be described by a power function. In the case of the 1-butanol GRM, the appearance of all measurements is curvilinear. It is postulated that the wettability of 1-butanol with glass is the reason for this behaviour. The strong contact of the drop with the glass prevented slippage to the top of the rod and realignment with the rotational symmetry axis. Consequently, the volume and surface area calculations were not precise for small drops.

There is a high initial volume flow in some curves, particularly in the 1-hexanol ALMs. The duration for degassing was shorter than usual in this instance. The residual gas caused a drop movement induced by the acoustic field, thereby increasing the dissolution. Subsequently, the gas was expelled by ultrasound, resulting in a dissolution behaviour of degassed drops of the same size. Some measurements exhibited an increasing slope towards the end of a measurement, indicating a decrease in the dissolution rate in an unusual manner. It is suspected that this behaviour was caused by non-water-soluble trace substances in the chemicals used. The residual volume contained these substances, which caused the dissolution rate to collapse (Figure 5, 1-butanol ALM).

The volume flow curves were surface interpolated linearly to 19 °C. At this temperature, the majority of the measurements were conducted and were within the temperature range of all measurement types. ${\dot{V}}_{\mathrm{inter}}\left(d_{A},T\;=\;19\;{}^{\circ}C\right)$ was then fitted by a power function combined with a linear term (Equation (2)), thereby defining the direction of mass transfer as dispersed to continuous (d→c):

(2) $${\dot{V}}_{\mathrm{fit}}\left(d_{A},T=19\;{}^{\circ}C\right)=a\cdot {\left(d_{A}\right)}^{n}+b\cdot d_{A},\;\mathrm{with}\;a,\;b\;>\;0\;\mathrm{and}\;n\;>\;1.$$

Small drops attached to the glass rod may have tended to tilt in a particular direction on the glass rod, compromising the symmetry regarding the z-axis. This may have led to an inaccuracy in the volume acquisition. Consequently, larger drops were more precise in terms of volume acquisition. Therefore, the weight factor of the data points for the fit was set to ${\left(d_{A}\right)}^{2}$, which gave greater attention to the larger dispersed volumes. The individual coefficients for the fit a,n,b, the coefficient of determination $R^{2}$ and the ranges of the diameter $d_{A}$, and the temperature *T* for Equation (2) are summarised in Table 2.

**Figure 6:**
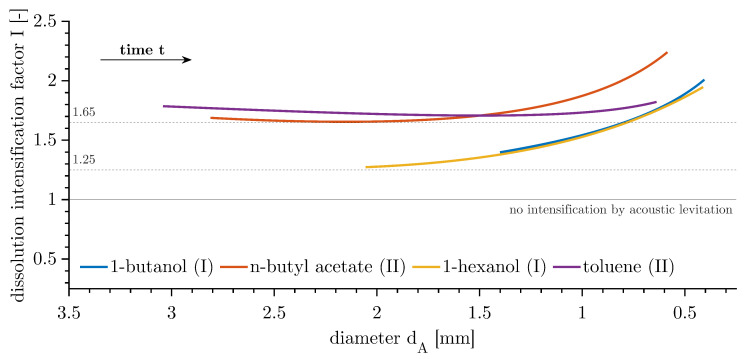
Intensification factor *I* for mass transfer of dissolving drops by acoustic levitation at a temperature of T= 19 °C, valid for the operating point and the methods described in [12].

The temperature interpolated and then the power function fitted trend of the volume flow ${\dot{V}}_{\mathrm{fit}}\left(d_{A}\right)$ allowed the definition of a dissolution intensification factor *I* for the chemical systems, as described in Equation (3). This intensification factor *I* compares the dissolution of the acoustically levitated drops with those observed in the glass rod measurements:

(3) $$I\left(d_{A}\right)=\frac{{\dot{V}}_{\mathrm{ALM},\;\mathrm{fit}}\left(d_{A}\right)}{{\dot{V}}_{\mathrm{GRM},\;\mathrm{fit}}\left(d_{A}\right)},$$

where $I(d_{A})$ is a function of the surface area-equivalent diameter $d_{A}$ and, therefore, compares drops of the same surface areas available for dissolution. Figure 6 illustrates the intensification effect caused by the use of acoustic levitation, valid for the presented low-impact acoustic levitation system and run with the chosen setting in the described operating point. The curves were only plotted for the overlap of the diameter $d_{A}$ of the ALM and GRM. It is important to note that alternations to the levitation setup, including changes to the power input, water level, or transducer horn immersion depth, as well as modifications to the degassing procedure, would inevitably lead to variations in the intensification factor *I*.

Although the dissolution times of the systems varied considerably, the four investigated cases exhibited a similar pattern in terms of the diameter $d_{A}$. The intensification factor *I* was >1 and started with a plateau before increasing with a decreasing diameter $d_{A}$. For case (I), where the convective plume could traverse the nodal plane without interruption, the plateau of the intensification factor began at approximately 1.25 at $d_{A}\approx$ 2.25 mm and increased with a decreasing diameter. Both systems, 1-butanol–water and 1-hexanol–water, exhibited congruent behaviour. For case (II), the plateau began at approximately 1.65 for n-butyl acetate–water. The toluene–water system displayed a slightly higher plateau and a lower gradient towards smaller diameters. The two types of behaviour can clearly be distinguished within Figure 6.

The standing waves interfere directly with an inhomogeneously dissolved substance in a continuous phase. The acoustic waves can directly affect single dissolved molecules or molecule clusters in a liquid and determine the path of propagation. It is suspected that there must be a measure to distinguish between the two types of behaviour, comparable to the acoustic contrast factor ϕ defined for dispersed drops in a continuous phase. However, this mechanism cannot locally enrich the concentration of the substance; it can only block the propagation through distinct barriers.

The dissolution intensification factor *I* is dependent on the type of acoustic behaviour, the size of the drop, and particularly, on the acoustic environment with a significant impact on the drop. The acoustic levitator was optimised for robust, low-impact operation by optimising the construction of the system, the point of operation, and the methods for liquid handling, namely, degassing.

It is postulated that the intensification factor *I* could be reduced even further towards I=1 by implementing minor adjustments in the operation of the acoustic levitator, e.g., by identifying the minimum required power input for the Langevin transducer to acoustically levitate a drop of a desired initial size. During the dissolution of the drop, its size decreases, allowing for a power input reduction. This type of operation could be optimised for a specific chemical system with its acoustic contrast factor ϕ. The disadvantage would be a prone operating point only valid for this specific chemical system, in which drops could potentially jump out of the system in the event of minor external influences.

## 4. Conclusions

In this study, the environment of a low-impact acoustic levitator, developed for investigations of single drops in mid-water, was experimentally investigated. The work focused on dissolution measurements and the intensification effect caused by acoustic levitation. The findings were intended to quantify the influence of acoustic levitation in more detail, thereby laying the foundation for future studies and avoiding unforeseen setbacks for other research groups.

As a basis for this study, the dissolving of immobilised drops of four chemical systems was studied in more detail to identify the dissolution intensification with two distinct experimental methods: acoustic levitation measurements (ALMs) and glass rod measurements (GRMs). The transient volume measurements were interpolated to a temperature T= 19 °C and then fitted by a power function combined with a linear term, to be able to make a general and consistent statement. Previous measurements with rainbow schlieren deflectometry indicated a difference in acoustic behaviour. The propagation of the convective plume in the continuous phase had two characteristics, which were confirmed by the evaluation of the dissolution measurements. Two distinct behaviours were identified, with a direct link to the dissolution intensification factor *I*. This factor exerted a significant influence on the mass transfer of inhomogeneously dissolved substances in the continuous phase:

case (I) The convective plume remained unaltered by the nodal plane of the standing wave used for acoustic levitation. This behaviour was investigated using 1-butanol–water and 1-hexanol–water. The spread was inhibited at locations with a maximum acoustic particle velocity of the acoustic field. A comparison between ALMs and GRMs indicated a minimum intensification factor of $I(d_{A}\;\geq \;2.25$ mm) ≈1.25$\left(\hat{=}25\%\right)$, which increased with the dissolution time and a decreasing diameter;
case (I) The convective plume of the chemical systems n-butyl acetate–water and toluene–water was deflected and held back by the nodal planes above and below the drop. The standing waves had the capacity to induce additional mass transfer in the opposite direction of natural convection. This resulted in an increased intensification factor of $I(d_{A}\geq$ 2 mm) ≈1.65$\left(\hat{=}65\%\right)$ as a minimum. With decreasing diameter, the intensification factor increased.

Acoustic levitation in liquid–liquid systems is a unique tool for the investigations of single drops, offering the potential for studies ranging from seconds to days. The main advantage is the optimal optical access for the precise volume acquisition of large drops, with uniform conditions and no interfering contact with any wall. However, it is essential to consider the influence of the acoustic field on the parameter of interest. In this study, the dissolution intensification factor *I* is dependent on the size of the drop ($d_{A}$) and is independent of the time scale of dissolution.

This technique could also be beneficial in other fields of fundamental research. The drops can be employed as microreactors for a variety of investigations, from biological to kinetic applications. This has already been practised in air for some time [17,18,19,20,21]. In studies on blood ageing, contact with walls leads to accelerated thrombus formation. In this instance, an inert continuous phase that is immiscible with blood must be identified and the acoustic levitator must be adapted to this material system. Another potential application could be the investigation of the degree of nanoparticle occupancy on drop surfaces, as the surface can be easily determined and observed here. These are only some examples of the types of applications that could be explored.

## Figures and Tables

**Table 1:** Physical properties of the used pure substances at 20 °C.

				Aqueous		Speed	Acoustic Contrast
		Manufacturer	Purity	Solubility	Density *	of Sound *	Factor ϕ * [12]
			[wt%]	[g/L]	[g/mL]	[m/s]	[-]
dispersed phase	1-butanol	ChemSolute	≥99.5	84 ^+^	0.80968	1256.41	−0.311
	n-butyl acetate	Supelco	≥99.5	6.8 ^+^	0.88147	1148.77	−0.338
	1-hexanol	ChemSolute	≥99.5	6.2 ^+^	0.81892	1320.72	−0.247
	toluene	Supelco	≥99.9	0.5 ^+^	0.86691	1320.34	−0.199
continuous phase	ultrapure water				0.99829	1482.46	0

* Own measurements with an Anton Paar Density and Sound Velocity Meter DSA 5000 M. ^+^ Data from [14], if necessary, are interpolated with a polynomial of the fourth degree to 20 °C.

**Table 2:** Coefficients for the fit of the volume flow ${\dot{V}}_{\mathrm{fit}}\left(d_{A}\right)$ in [µL/h] for T = 19 °C.

	Coefficients for Equation (2)				Interpolation Ranges	
	a	n	b	$R^{2}$	$d_{A}$ [mm]	T [°C]
1-butanol (ALM)	22.07	1.977	1.589 $\times \;10^{-5}$	0.999	0.34–3.91	19.0–19.5
1-butanol (GRM)	14.31	2.27	1.596 $\times \;10^{-7}$	0.975	0.40–1.40	19.1–19.7
n-butyl acetate (ALM)	0.07724	2.633	7.284 $\times \;10^{-1}$	0.997	0.33–5.29	19.3–23.5
n-butyl acetate (GRM)	0.4301	1.442	2.798 $\times \;10^{-8}$	0.998	0.58–2.81	15.3–19.7
1-hexanol (ALM)	0.05027	3.227	7.471 $\times \;10^{-1}$	0.975	0.33–5.14	18.4–20.8
1-hexanol (GRM)	0.2157	2.102	3.062 $\times \;10^{-1}$	0.985	0.41–2.06	17.6–20.5
toluene (ALM)	0.02701	1.855	3.553 $\times \;10^{-2}$	0.999	0.37–2.97	22.1–22.8
toluene (GRM)	0.0361	1.442	5.015 $\times \;10^{-7}$	0.997	0.61–3.04	17.8–19.2

## Data Availability

The data that support the findings of this study are available on request.

## Supplementary Materials

The following supporting information can be downloaded at: https://www.mdpi.com/article/10.3390/mi15060805/s1, Video S1: Acoustic levitation measurement—time-lapse of a dissolving n-butyl acetate drop in water; Video S2: Glass rod measurement—time-lapse of a dissolving n-butyl acetate drop in water; Video S3: Rainbow schlieren deflectometry measurement—case (I)—dissolving 1-butanol drop in water, as in [12]; Video S4: Rainbow schlieren deflectometry measurement—case (II)—dissolving morpholine–toluene drop in water with morpholine as the transferring component.

## Abbreviations

The following abbreviations are used in this manuscript:

ALM	acoustic levitation measurement
GRM	glass rod measurement
RSD	rainbow schlieren deflectometry
c	continuous
d	dispersed
exp	experimental
fit	fitted
inter	interpolated
max	maximum

## References

[1] Basu S., Rao D.C.K., Chattopadhyay A., Chakraborty J. (2021). Dissolution dynamics of a vertically confined sessile droplet. Phys. Rev. E.

[2] Chong K.L., Li Y., Ng C.S., Verzicco R., Lohse D. (2020). Convection-dominated dissolution for single and multiple immersed sessile droplets. J. Fluid Mech..

[3] Dietrich E., Wildeman S., Visser C.W., Hofhuis K., Kooij E.S., Zandvliet H.J.W., Lohse D. (2016). Role of natural convection in the dissolution of sessile droplets. J. Fluid Mech..

[4] Su J.T., Needham D. (2013). Mass Transfer in the Dissolution of a Multicomponent Liquid Droplet in an Immiscible Liquid Environment. Langmuir.

[5] Heine J.S., Bart H.J. (2019). Visualization of Mass Transfer during Droplet Formation. Chem. Eng. Technol..

[6] Wegener M., Paschedag A., Kraume M. (2009). Mass transfer enhancement through Marangoni instabilities during single drop formation. Int. J. Heat Mass Transf..

[7] Javadi A., Bastani D., Taeibi-Rahni M. (2005). Mass transfer during drop formation on the nozzle: New flow expansion model. AIChE J..

[8] Heine J.S., Bart H. (2019). Local analysis of Marangoni effects during and after droplet formation. Can. J. Chem. Eng..

[9] Arendt B., Eggers R. (2007). Interaction of Marangoni convection with mass transfer effects at droplets. Int. J. Heat Mass Transf..

[10] Agble D., Mendes-Tatsis M. (2000). The effect of surfactants on interfacial mass transfer in binary liquid–liquid systems. Int. J. Heat Mass Transf..

[11] Heine J.S., Schulz J.M., Junne H., Böhm L., Kraume M., Bart H. (2020). Real-Time Visualization of Internal and External Concentration Fields in Multiphase Systems via Laser-induced Fluorescence and RSD During and After Droplet Production. Chem. Ing. Tech..

[12] Ruiken J.P., Villwock J., Kraume M. (2023). Behaviour of Acoustically Levitated Drops in Mid-Water. Micromachines.

[13] Misek T., Berger R., Schröter J. (1985). Standard test systems for liquid extraction. Institution of Chemical Engineers.

[14] Rumble J. (2023). CRC Handbook of Chemistry and Physics.

[15] Schulz J.M., Junne H., Böhm L., Kraume M. (2020). Measuring local heat transfer by application of Rainbow Schlieren Deflectometry in case of different symmetric conditions. Exp. Therm. Fluid Sci..

[16] Junne H., Jurtz N., Schulz J.M., Kraume M., Böhm L. (2023). Rainbow Schlieren Deflectometry for spherical fields: A new algorithm and numerical validation approach. Numerical Heat Transfer, Part B: Fundamentals.

[17] Santesson S., Andersson M., Degerman E., Johansson T., Nilsson J., Nilsson S. (2000). Airborne Cell Analysis. Anal. Chem..

[18] Omrane A., Santesson S., Aldén M., Nilsson S. (2004). Laser techniques in acoustically levitated micro droplets. Lab Chip.

[19] Westphall M.S., Jorabchi K., Smith L.M. (2008). Mass Spectrometry of Acoustically Levitated Droplets. Anal. Chem..

[20] Scheeline A., Behrens R.L. (2012). Potential of levitated drops to serve as microreactors for biophysical measurements. Biophys. Chem..

[21] van Wasen S., You Y., Beck S., Riedel J., Volmer D.A. (2021). Quantitative Analysis of Pharmaceutical Drugs Using a Combination of Acoustic Levitation and High Resolution Mass Spectrometry. Anal. Chem..

